# Kv3.1b and Kv3.3 channel subunit expression in murine spinal dorsal horn GABAergic interneurones

**DOI:** 10.1016/j.jchemneu.2011.02.003

**Published:** 2011-09

**Authors:** A. Nowak, H.R. Mathieson, R.J. Chapman, G. Janzsó, Y. Yanagawa, K. Obata, G. Szabo, A.E. King

**Affiliations:** a Institute of Membrane and Systems Biology, Faculty of Biological Sciences, University of Leeds, Leeds, LS2 9JT, UK; b Department of Anatomy and Histology, Faculty of Veterinary Science, Szent István University, Budapest, Hungary; c Department of Genetic and Behavioral Neuroscience, Gunma University Graduate School of Medicine, Maebashi, Gunma 371-8511, Japan; d Department of Gene Technology and Developmental Neurobiology, Institute of Experimental Medicine, H-1083 Budapest, Hungary

**Keywords:** GAD65, GAD67, Immunohistochemistry, c-*fos*, Capsaicin, Nociception, Potassium channel

## Abstract

GABAergic interneurones, including those within spinal dorsal horn, contain one of the two isoforms of the synthesizing enzyme glutamate decarboxylase (GAD), either GAD65 or GAD67. The physiological significance of these two GABAergic phenotypes is unknown but a more detailed anatomical and functional characterization may help resolve this issue. In this study, two transgenic Green Fluorescent Protein (GFP) knock-in murine lines, namely GAD65-GFP and GAD67-GFP (Δneo) mice, were used to profile expression of *Shaw*-related Kv3.1b and Kv3.3 K^+^-channel subunits in dorsal horn interneurones. Neuronal expression of these subunits confers specific biophysical characteristic referred to as ‘fast-spiking’. Immuno-labelling for Kv3.1b or Kv3.3 revealed the presence of both of these subunits across the dorsal horn, most abundantly in laminae I–III. Co-localization studies in transgenic mice indicated that Kv3.1b but not Kv3.3 was associated with GAD65-GFP and GAD67-GFP immunopositive neurones. For comparison the distributions of Kv4.2 and Kv4.3 K^+^-channel subunits which are linked to an excitatory neuronal phenotype were characterized. No co-localization was found between GAD-GFP +ve neurones and Kv4.2 or Kv4.3. In functional studies to evaluate whether either GABAergic population is activated by noxious stimulation, hindpaw intradermal injection of capsaicin followed by c-*fos* quantification in dorsal horn revealed co-expression c-*fos* and GAD65-GFP (quantified as 20–30% of GFP +ve population). Co-expression was also detected for GAD67-GFP +ve neurones and capsaicin-induced c-*fos* but at a much reduced level of 4–5%. These data suggest that whilst both GAD65-GFP and GAD67-GFP +ve neurones express Kv3.1b and therefore may share certain biophysical traits, their responses to peripheral noxious stimulation are distinct.

## Highlights

► Kv3.1b and Kv3.3 expression was studied in dorsal horn GABAergic interneurones. ► Kv3.1b or Kv3.3 was most abundantly expressed in laminae I–III. ► Kv3.1b but not Kv3.3 was associated with GAD65 and GAD67 neurones. ► Capsaicin-induced c-fos expression was localized mainly to GAD65-GFP interneurones.

## 1 Introduction

Glutamate decarboxylase (GAD) synthesizes GABA, the main inhibitory neurotransmitter in the adult central nervous system, and exists as two isoforms, named GAD65 or GAD67 on the basis of their respective molecular weights. Each isoform is expressed in different amounts within GABAergic interneurones and although the physiological significance of this difference is not established, it has been suggested to be related to factors such as afferent terminal GABA release mechanisms or ‘tonic’ versus ‘phasic’ firing properties of single neurones (Soghomonian and Martin, 1998). In the rat dorsal horn (DH) GAD65 immunoreactivity is abundant in superficial laminae with decreasing amounts localized to the deeper DH laminae (Mackie et al., 2003). GAD67 immunoreactive profiles are concentrated within laminae I–III with moderate amounts also in laminae IV–VI (Mackie et al., 2003). These data on spinal GAD65 and GAD67 infer a heterogeneous distribution of GABAergic neurones in the DH with the highest numbers localized to laminae I–III, an area that is richly innervated by nociceptive sensory afferents. It is estimated that about 30% of neurones in LI–II are GABAergic (Todd and Sullivan, 1990) and these can induce either pre- or postsynaptic inhibition of primary afferents (Barber et al., 1978). In DH, there appears to be specific patterns of postsynaptic targeting by classes of GABAergic neurones (Puskar et al., 2001), this presumably shaping the output to other laminae or projection pathways. GABA_A_ and GABA_B_ receptors are localized to primary afferents where they act to modulate glutamate and peptidergic transmitter release (Malcangio and Bowery, 1996). Inhibitory neurones in DH are activated by noxious stimuli, as evidenced by enhanced expression of the immediate early gene c-*fos* in GABA-immunoreactive neurones (Todd et al., 1994) but it is unknown as to whether this is mainly within the GAD65 or GAD67 interneurone population.

Phenotypically distinct populations of GABAergic interneurones have been characterized in some detail for both brain and spinal cord (Todd and Spike, 1993; Kawaguchi et al., 1995). The functional importance of anatomical and biophysical heterogeneities is emerging from data that reveal specific roles for GABAergic interneuronal subtypes within the circuitry that they are embedded (McBain and Fisahn, 2001). For example, in cortical and hippocampal networks GABAergic interneurones with a fast-spiking phenotype are crucially involved in detection and promotion of synchronous activity (Galarreta and Hestrin, 2001). As inferred by the name, fast-spiking cells are able to maintain high frequency firing with little evident adaptation (Kawaguchi, 2001). This striking biophysical characteristic is due to the expression of K^+^-channel subunits belonging to the *Shaw*-related Kv3 family (Rudy et al., 1999). The expression profiles of Kv3 subunits in either GAD65 or GAD67 populations of GABAergic interneurones in the DH are currently unknown although it is known that Kv3.1b and Kv3.3 but not Kv3.2 are localized to DH somata (Deuchars et al., 2001; Brooke et al., 2006). A more complete phenotypic characterization of GAD65 compared to GAD67 interneurone populations, in particular the expression of Kv3 subunits, will provide insight into their possible function within DH circuitry.

In this study, we have used two transgenic Green Fluorescent Protein (GFP) knock-in mouse lines, namely GAD65-GFP and GAD67-GFP (Δneo) knock-in mice, to profile expression of Kv3 subunits in identified populations of GABAergic DH interneurones. For comparison purposes, we determined the expression levels of the *shal*-related K^+^ channel subunits Kv4.2 and Kv4.3 which are also localized to DH but putatively within a subset of excitatory interneurones (Hu et al., 2006; Huang et al., 2005). To evaluate whether there is activation of either of these two classes of GABAergic interneurones following peripheral noxious stimulation, we used paw intradermal injection of capsaicin followed by the quantification of nuclear c-*fos* expression in DH.

## 2 Materials and methods

### 2.1 Animals

All procedures were carried out in accordance with the UK Animals (Scientific Procedures) Act of 1986 and experimental protocols were approved by the local Faculty of Biological Sciences Ethics Committee. Experiments utilized mainly either GAD65-GFP or GAD67-GFP (Δneo) mice bred as heterozygous strains (due to the lethality of homozygotes) on a C57BL6 background (Harlan, UK). In some studies, C57BL6 GAD65-GFP or GAD67-GFP −/− littermates were used. The generation and characterization of the GAD67-GFP (Δneo) knock-in mouse has been described previously (Tamamaki et al., 2003; Tsunekawa et al., 2005) but briefly, GFP cDNA was targeted to the GAD67 locus and GFP expressed under the control of the endogenous GAD67 promoter. Similarly, GAD65-GFP mice expressed GFP under the control of the GAD65 gene promoter (Lopez-Bendito et al., 2004; Labrakakis et al., 2009). Neonatal mice were phenotyped by the observation of GFP fluorescence in the intact brains of mice exposed to ultra-violet illumination.

### 2.2 Kv subunit immunohistochemistry

Adult mice were deeply anaesthetised with urethane (2 g/kg, i.p.) and trans-cardially perfused with fixative containing 4% paraformaldehyde (PFA; in 0.1 M phosphate buffer, pH 7.4). The spinal cords were removed and post-fixed in 4% PFA overnight at 4 °C. Transverse sections (50 μm) from lumbar spinal cord were cut on a Vibratome (Leica, Milton Keynes, UK) and collected into phosphate buffered saline (PBS; pH 7.2). Sections were permeabilised by the inclusion of 0.1% Triton X-100 (Sigma, UK) in the primary antibody solution, washed in PBS (3 × 10 min) and transferred to either anti-Kv3.1b (Alomone Labs, rabbit polyclonal, 1:1000), anti-Kv3.3 (Alomone Labs, rabbit polyclonal, 1:1000), anti-Kv4.2 (NeuroMab, mouse monoclonal, 1:100) or anti-Kv4.3 (NeuroMab, mouse monoclonal, 1:100) primary antibodies for 12–36 h at 4 °C. Prior to secondary antibody application, all sections were washed in PBS (3 × 10 min). Anti-Kv3.1b and anti-Kv3.3 were visualised using Alexa^488^-conjugated donkey anti-rabbit (1:1000, Invitrogen) or using Cy3-conjugated anti-rabbit (1:1000; Jackson ImmunoResearch Laboratories). Anti-Kv4.2 and anti-Kv4.3 were visualised using Alexa^488^-conjugated donkey anti-mouse (1:1000, Invitrogen) or using Cy3-conjugated anti-mouse (1:1000, Jackson ImmunoResearch Laboratories). Secondary antibodies were applied for 2–3 h (4 °C) and sections washed in PBS before drying, mounting and cover slipping using Vectashield (Vector laboratories, Peterborough, UK). As a procedural control for antiserum specificity, primary antibodies were replaced by PBS and under these conditions no staining occurred. The % co-localization of Kv3.1b and GAD65-GFP or GAD67-GFP was calculated from the total number GFP-immunopositive cells, counted in non-consecutive longitudinal lumbar sections, relative to the number of neurones that were surrounded by Kv3.1b puncta.

### 2.3 c-*fos* immunohistochemistry and quantification

To evaluate c-*fos* expression in response to peripheral noxious stimuli, adult GAD65-GFP or GAD67-GFP mice were deeply anaesthetised with urethane (2 g/kg, i.p.) and following the loss of the paw withdrawal reflex, capsaicin (3%, 10 μl) was injected into the intraplantar surface of one hind paw. For comparison, capsaicin was also injected into one paw of C57BL6 GAD65-GFP or GAD67-GFP −/− littermates and in a separate control group (*n* = 3) only saline (10 μl) was injected into the paw. Animals were maintained under light anaesthesia for 45–60 min before being trans-cardially perfused with fixative containing 4% paraformaldehyde (PFA; in 0.1 M phosphate buffer, pH 7.4). This end-point was selected on the basis of published literature indicating that c-*fos* levels in superficial laminae peak at this time (Hunt et al., 1987). The spinal cords were removed and post-fixed in 4% PFA overnight at 4 °C. Longitudinal and transverse sections (50 μm) from the lumbar region (L3–L6) of the spinal cord were cut on a Vibratome (Leica, Milton Keynes, UK) and collected into PBS (pH 7.2). Sections were permeabilised by the inclusion of 0.1% Triton X-100 (Sigma, UK) in the primary antibody solution, washed in PBS (3 × 10 min) and transferred to anti-c-*fos* (Oncogene, rabbit polyclonal, 1:10,000) for 12–36 h at 4 °C. Prior to secondary antibody application, all sections were washed in PBS (3 × 10 min). Anti-c-*fos* was visualised using Cy3-conjugated anti-rabbit (1:1000, Jackson ImmunoResearch Laboratories). Secondary antibodies were applied for 2–3 h (4 °C) and sections washed in PBS before drying, mounting and cover slipping using Vectashield (Vector Laboratories, Peterborough, UK). As a control to ensure that the antiserum detected the appropriate antigen, replacement of the primary antibodies with PBS eliminated immuno-staining.

For each animal, non-consecutive longitudinal sections (minimum = 6) from lumbar spinal cord, ipsilateral and contralateral to the site of capsaicin injection were randomly selected for nuclear c-*fos* counts. Counts from each section were taken from an equal-sized region (650 μm^2^) across both the superficial and deep dorsal horn under 40× microscopic magnification. Total cell counts were calculated for both superficial (laminae I/II) and deep (laminae III–VI) DH in both ipsilateral and contralateral sections. All data sets were tested for normality before continuing with a *t*-test, or for comparison of multiple data sets, one-way ANOVA (Minitab version 13). Data are expressed as means ± standard error of the mean (S.E.M.) where *n* represents the number of animals. The % co-localisation of c-*fos* and GAD65-GFP or GAD67-GFP cells was calculated according to the number of GFP immunoreactive cells relative to the number of c-*fos* expressing cells.

### 2.4 Image capture and manipulation

Low- and high-power images were captured using a Nikon (Surrey, UK) Eclipse E600 epifluorescence microscope and AcQuis image capture software (Synoptics, Cambridge, UK). For figure production, CorelDraw13 software was used to adjust brightness, contrast and intensity, if appropriate.

## 3 Results

### 3.1 Distribution of GAD65-GFP and GAD67-GFP expression in murine lumbar spinal cord

**Fig. 1:**
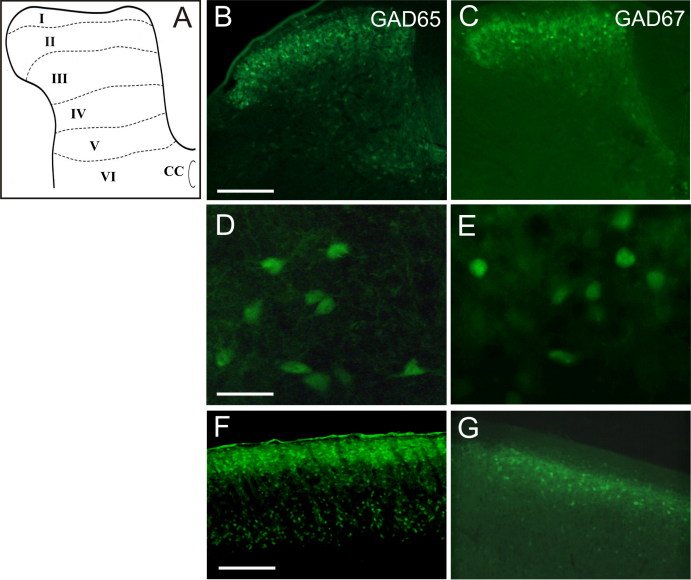
Distribution of GAD65-GFP (B, D and F) and GAD67 GFP (C, E and G) +ve neurones in mouse dorsal horn. (A) Schematic representation of dorsal horn laminae indicating approximate boundaries of laminae I–VI. CC, central canal. (B and C) Transverse section of mouse spinal cord revealing abundant GAD65- and GAD67-associated GFP immunofluorescence in dorsal horn superficial laminae, especially across lamina IIi. (D and E) High magnification image illustrating examples of cell bodies and unidentified fibre processes +ve for GFP immunfluorescence. (F and G) Longitudinal sections indicate a near uniform distribution of GFP +ve neurones within L3–L6 of lumbar spinal cord. Scale bars: B, C, F and G, 200 μm; D, E, 20 μm.

In both GAD65-GFP and GAD67-GFP (Δneo) transgenic mouse tissues, GFP +ve neurones were localized throughout the DH but were particularly abundant in the superficial (I–II) compared to deep laminae (III–VI) (Fig. 1B and C). For each mouse strain, GFP fluorescence was observed in both unidentified processes and somata of presumed GABAergic interneurones (Fig. 1D and E). For GAD65-GFP mice, the highest density of GFP +ve fluorescence was within laminae I–III with more moderate expression scattered throughout the deeper laminae IV–VI (Fig. 1B, D and F) and especially within the medial aspect of laminae VI. Within the superficial DH, there was a distinctly strong band of GFP +ve cells within lamina I and the inner region of lamina II (IIi). For GAD67-GFP (Δneo) mice, GFP +ve profiles were also numerous throughout laminae I–III and were particularly abundant within laminae I and IIi (Fig. 1C, E and G). GAD67 GFP +ve profiles were localized to deeper laminae IV–VI, particularly the medial aspect of these laminae but the overall expression levels in these deeper laminae was considerably reduced compared to laminae I and LII. Visualization of GFP +ve neurons in longitudinal sections of lumbar spinal cord (L3–L6) revealed a uniform rostro-caudal distribution (Fig. 1F and G).

### 3.2 Distribution of Kv3 and Kv4 subunits in lumbar spinal cord of GAD65-GFP and GAD67-GFP (Δneo) mice

**Fig. 2:**
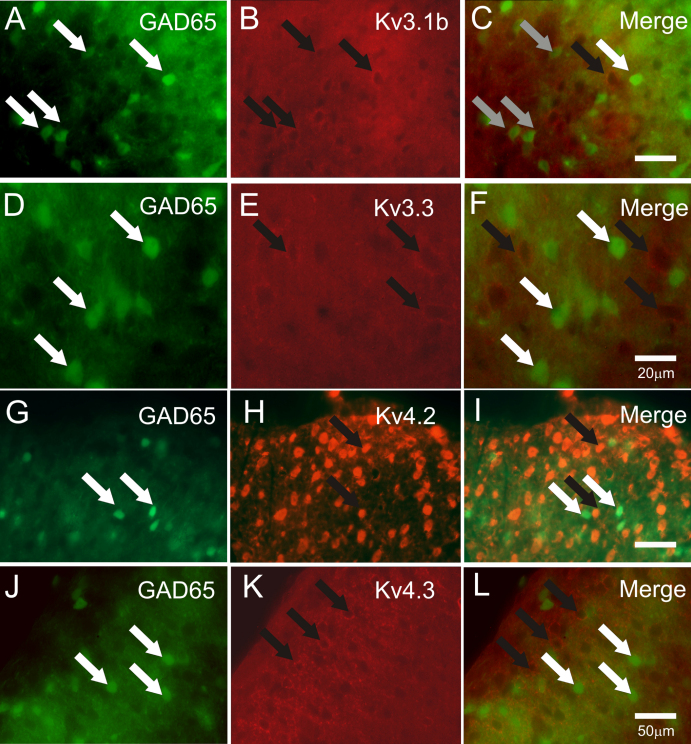
Expression of Kv3 and Kv4 subunits in GAD65-GFP mouse dorsal horn. (A–C) GFP +ve neurones (A) and Kv3.1b subunit immunfluorescence (B) localized to superficial dorsal horn. Merged images (C) indicate a significant overlap between GFP +ve neurones and Kv3.1b immunoreactivity (grey arrows) although examples exist where GFP fluorescence (white arrows, green) and Kv3.1b fluorescence (black arrows, red) are clearly not co-localized. (D–F) GFP +ve neurones (D) and Kv3.3 subunit immunofluorescence (E) localized to superficial dorsal horn. Merged images (C) indicate no significant overlap between GFP +ve neurones (white arrows, green and Kv3.3 immunoreactivity (black arrows, red). (G–I) GFP +ve neurones (G) and Kv4.2 immunoreactivity (H) localized to superficial dorsal horn. Merged images (I) indicate no association between GFP fluorescence (white arrows, green) and immunoreactivity for Kv4.2 (back arrows, red). (J–L) Merged images for GAD 65 +ve neurones (J, green) and Kv 4.3 (K, red) indicate expression by separate populations. Scale bars: A–F, 20 μm; G–L, 50 μm.

**Fig. 3:**
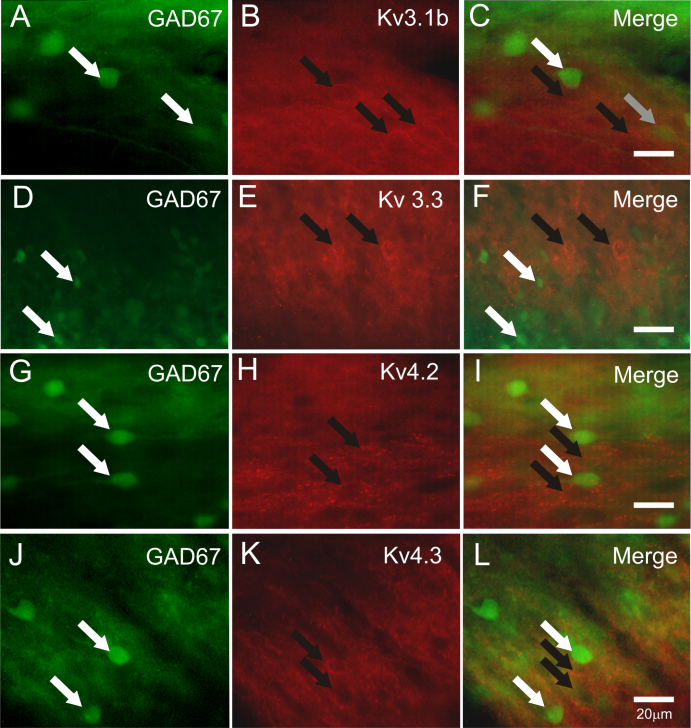
Expression of Kv3 and Kv4 subunits in GAD67-GFP (Δneo) mouse dorsal horn. (A–C) GFP +ve neurones (A) and Kv3.1b subunit immunfluorescence (B) localized to superficial dorsal horn. Merged images (C) indicate that some GFP +ve neurones also express Kv3.1b immunoreactivity (grey arrows) although examples exist where GFP fluorescence (white arrows, green) and Kv3.1b fluorescence (black arrows, red) are not co-localized. (D–F) GFP +ve neurones (D) and Kv3.3 subunit immunofluorescence (E) localized to superficial dorsal horn. Merged images (C) indicate no significant overlap between GFP +ve neurones (white arrows, green and Kv3.3 immunoreactivity (black arrows, red). (G–H) GFP +ve neurones (G) and Kv4.2 immunoreactivity (H) localized to superficial dorsal horn. (J–K) GFP +ve neurones (J) and Kv4.2 immunoreactivity (K) localized to superficial dorsal horn. Merged images (I and L) indicate no association between GFP fluorescence (white arrows, green) and immunoreactivity for Kv4.2 or Kv4.3 (black arrows, red). Scale bars all 20 μm.

To determine the extent to which Kv3 or Kv4 subunits were expressed in GABAergic neurons with either a GAD65- or GAD67-GFP phenotype, immuno-labelling for Kv3 and Kv4 was performed using spinal cords from GAD65-GFP (Fig. 2) and GAD67-GFP (Δneo) (Fig. 3) mice. In both GFP reporter mouse strains, the distribution of Kv3 and Kv4 receptor subunits in the DH was similar to that observed in GFP −/−ve litter mates.

Strong immunofluorescence for Kv3.1b or Kv3.3 subunits, associated with both somata and fibres, was detected throughout the DH of the lumbar spinal cords of GAD65-GFP and GAD67-GFP (Δneo) mice. In both mouse strains, immuno-labelling for these Kv3 subunits appeared as punctate fluorescence surrounding somata and in some instances labelling extended along fibres, presumed to be axons or dendrites, emanating from the labelled cell body (Figs. 2A–F and 3A–F). For Kv3.1b, immunoreactivity was intense across laminae I–III and was closely associated with numerous cell bodies within this region (Figs. 2B and 3B). More moderate immuno-labelling for Kv3.1b was evident in deeper laminae (not illustrated). For Kv3.3 subunits, immunoreactivity was observed within fibres of the neuropil surrounding neurons and as distinct puncta closely associated with somata within laminae I–III (Figs. 2E and 3E). Kv3.3 immuno-positive structures were observed also within deep DH laminae (not illustrated).

In comparing the distribution of immunoreactivity for Kv3.1b subunits and GAD65-GFP +ve neurones, whilst significant numbers of Kv3.1b immunoreactive profiles were not GFP +ve, a proportion of GAD65-GFP neurons were surrounded by Kv3.1b immunoreactive puncta (Fig. 2A–C). However, not all GAD65-GFP +ve somata were associated with Kv3.1b immunoreactivity. Quantification of Kv3.1b and GAD65-GFP co-localization indicated a co-expression level of 25% (278/1109 neurones, *n* = 3). Similarly, a proportion of GAD67-GFP +ve somata were associated with Kv3.1b immunoreactive puncta (Fig. 3A–C) and Kv3.1b profiles that did not associate with GFP were observed. Quantification of Kv3.1b and GAD67-GFP co-localization indicated a co-expression level of 31% (634/2039 neurones, *n* = 4). The % co-localization values calculated for Kv3.1b in the two mouse strains were not significantly different. Whilst Kv3.3. immunoreactivity was observed in the DH of both strains there was no clear association between GFP +ve and Kv3.3 immunoreactive profiles (Figs. 2D–F and 3D–F).

In contrast to this rather dispersed Kv3 immunoreactivity distribution, the pattern for Kv4 subunits was more restricted with immunoreactive structures for either Kv4.2 or Kv4.3 predominantly in laminae I–III, with particularly strong immunoreactivity in lamina II. Kv4.2 immunoreactivity was observed either as intense labelling across numerous cell somata (Fig. 2H) or as distinct puncta tightly surrounding cell bodies (Fig. 3H). The latter punctate profile of immunolabelling typified Kv4.3 (Figs. 2K and 3K) immunoreactivity, as was found for Kv3 immunolabelling. For GAD65-GFP (Fig. 2G–L) and GAD67-GFP (Δneo) mice (Fig. 3G–L), no association could be established between GFP +ve cells and Kv4.2 or 4.3 subunit expression in the DH.

### 3.3 Nociceptive-induced c-*fos* expression in lumbar spinal cord of GAD65-GFP and GAD67-GFP (Δneo) mice

**Fig. 4:**
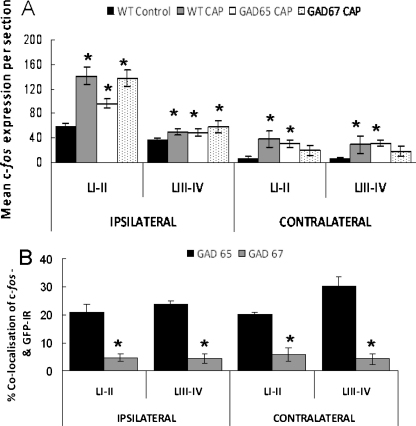
Quantified data for the expression of capsaicin-induced c-*fos* in mouse dorsal horn. (A) c-*fos* counts in ipsilateral and contralateral dorsal horn for wild-type (WT) (*n* = 3), GAD65-GFP (*n* = 6) and GAD67-GFP (Δneo) (*n* = 5) mice. Data are presented for superficial (I–II) and deep (III–VI) laminae and values represent the mean cell counts for c-*fos* expression per section (asterisks represent statistical significance of data as compared to WT controls that received intradermal saline injection, *n* = 3, *P* < 0.05). (B) Levels of % co-localization for GAD65-GFP and GAD67-GFP c-*fos* +ve neurones in superficial and deep dorsal horns of ipsi- and contralateral spinal cord (asterisks indicate statistically significant differences between co-expression levels in the two GAD-GFP mouse strains, *P* < 0.05).

To determine whether GAD65- or GAD67-containing GABAergic interneurones were reactive to peripheral noxious stimulation, the extent to which c-*fos* co-localized with GFP +ve neurons after hindpaw injection of capsaicin was examined for the two transgenic strains. In control mice (GAD65- or GAD67-GFP (Δneo) −/− littermates) that received only a saline injection, modest c-*fos* immunoreactivity was observed in superficial and deep DH laminae on the ipsilateral side of the spinal cord with the highest counts associated with laminae I–II (mean cell counts: 59 ± 6 cells in laminae I–II; 36 ± 4 cells in laminae III–IV; Fig. 4A, *n* = 3). Low levels (<10 cells) of c-*fos* expressing cells were evident in superficial and deep laminae contralateral to saline injection (Fig. 4A). In wild-type (GAD65- or GAD67-GFP −/−) littermates), intradermal capsaicin-injection increased the number of c-*fos*-expressing cells significantly both ipsilaterally and contralaterally (Fig. 4A). However, the superficial laminae of the ipsilateral spinal cord showed the greatest augmentation of c-*fos* immunoreactivity rising by 140% compared to the saline-injected control group (mean cell count: 141.3 ± 14.4 cells in laminae I–II; 50 ± 5 cells or 38% in laminae III–IV; Fig. 4A, *P* < 0.05, *n* = 3). In both strains of GAD-GFP mice, after capsaicin injection the highest levels of c-*fos* expression were in ipsilateral superficial laminae. Quantified data for GAD65-GFP mice (Fig. 4A) reveal a mean cell count of 97 ± 5 (*n* = 6) which compared to the control saline-injected group represents an increase of 64% for GAD65-GFP mice. For GAD67-GFP (Δneo) transgenic mice, the ipsilateral laminae I–II mean cell count following capsaicin was 138 ± 13.2, representing a 134% rise in the number of c-*fos* expressing neurons (Fig. 4A, *P* < 0.05, *n* = 5). For both strains of GAD-GFP mice, smaller but still significant capsaicin-induced increases in c-*fos* were quantified for ipsilateral deep laminae. In contralateral spinal cord, the overall expression levels of c-*fos* were much reduced compared to the ipsilateral cord and in GAD65-GFP but not GAD67-GFP (Δneo) mice counts were significantly increased by intradermal capsaicin.

**Fig. 5:**
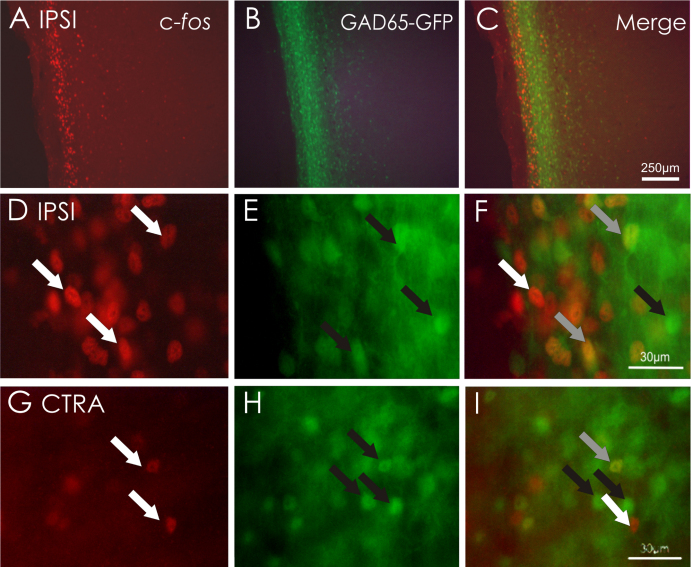
Expression of c-*fos* in GAD65-GFP +ve neurones localized to mouse dorsal horn. (A–C) Longitudinal section of dorsal horn illustrating distribution of c-*fos* (A, red) and GAD65 +ve neurones (B, green) within ipsilateral spinal cord. Merged images (C) indicate a degree of overlap in the distribution of GAD65-GFP +ve neurones and c-*fos*. (D–F) High magnification examples showing single ipsilateral DH neurones in which both c-*fos* (D, white arrows) and GAD65-GFP (E, black arrows) are co-localized (F, grey arrows). (G–I) High magnification examples showing single contralateral DH neurones in which both c-*fos* (G, white arrows) and GAD65-GFP (H, black arrows) are co-localized (I, grey arrows). Note that not all GAD65-GFP neurones express c-*fos* and conversely, a significant proportion of c-*fos* labelled neurones are not GAD65-GFP +ve.

In longitudinal sections of dorsal horn, c-*fos* immunoreactivity appears as a distinct band localized to superficial dorsal horn (Fig. 5A). With respect to co-localization of c-*fos* and GAD65- or GAD67-GFP +ve neurons, this was evident at modest levels (Fig. 5C, F and I). Co-expression of GAD65-GFP and c-*fos* was observed within superficial and deep laminae of both ipsi- and contralateral DHs at quantified levels of between 20 and 30% (Fig. 4B). In longitudinal sections from GAD65-GFP mice treated with capsaicin (Fig. 5), many neurons were c-*fos* immunopositive but GFP −ve indicating significant activation of an alternative non-GABAergic neuronal phenotype. Conversely, a proportion of GAD65-GFP +ve neurons were immuno-negative for c-*fos* (Fig. 5) suggesting only partial recruitment of this neuronal phenotype by peripheral chemical nociception. For GAD67-GFP mice, there was little overlap between GFP +ve neurons and the c-*fos* immunopositive population (not illustrated). Quantified data indicate a GAD67-GFP and c-*fos* co-localization levels of 4–5% both ipsi- and contralateral. Data values calculated for GAD67-GFP and c-*fos* were significantly lower than values quantified for GAD65-GFP (Fig. 4B).

## 4 Discussion

The use of two GAD-GFP reported mouse strains in this study has confirmed the presence of distinct populations of GAD65- and GAD67-containing interneurones within the murine lumbar DH. Both GAD isoforms were particularly abundant in superficial laminae I–III although expression was not exclusively restricted to these laminae since GFP +ve profiles were scattered, albeit at much lower densities, across laminae III–VI. These data accord with the distribution pattern reported for the rat spinal cord on the basis of immunocytochemical studies that also revealed co-localization of the neuronal glycine transporter GLYT2 in GAD +ve boutons (Mackie et al., 2003). The expression of GFP in superficial laminae of the GAD67-GFP (Δneo) mouse strain resembles that reported for another equivalent transgenic mouse line, referred to as the GIN (GFP-expressing inhibitory neurones) mouse (Heinke et al., 2004) and the concentrated superficial DH distribution of GAD65-GFP has also been described (Hughes et al., 2005; Labrakakis et al., 2009). GAD-GFP expressing neurones in transgenic mouse strains have been confirmed as GABAergic (Makinae et al., 2000; Heinke et al., 2004; Huang et al., 2008). Further analysis of the neurochemical phenotype of GAD67-GFP neurones revealed that they constitute a heterogeneous group differentially co-expressing parvalbumin, glycine but not PKCγ (Heinke et al., 2004; Dougherty et al., 2009).

Analysis of the distribution of the expression of Kv4 potassium subunits in the murine DH indicated a profile of expression similar to that reported for adult rat DH. Kv4.2 and Kv4.3 subunit expression was localized to superficial laminae with no evident co-expression in either GAD65-GFP or GAD67-GFP +ve neurones, a finding that is consistent with the reported presence of Kv4 in a population of μ-opioid receptor +ve excitatory interneurones within lamina II (Huang et al., 2005). As has been reported for rat sensory neurones (Huang et al., 2005), in some dorsal horn neurones cytoplasmic rather than cell-surface Kv4.2 subunit expression was observed. Trafficking of Kv4 receptor subunits from the cytoplasmic domain to the membrane surface is promoted by several factors, e.g. Kv channel-interacting proteins (KChIPs) and potassium channel accessory protein (KChAP), it is suggested to be one mechanism of activity-induced neuroplasticity. Kv4.2 mediates the majority of A-type potassium currents in dorsal horn and an increase in A-type currents would be predicted to reduce overall neuronal excitability (Hu et al., 2006).

In rat thoracic spinal cord, Kv3.1b has been localized to cell bodies and fibres within superficial and deep DH (Deuchars et al., 2001) whilst Kv3.3 is expressed more sparsely in laminae I–III compared to laminae IV/V (Brooke et al., 2006). In this study, Kv3.1b and Kv3.3 subunits were found across the mouse DH and immuno-labelling was associated with somata and unidentified fibres. Expression of Kv3.1b was observed in proximity to GAD65-GFP or GAD67-GFP +ve neurones in the transgenic mouse strains whereas Kv3.3 immunoreactivity and GFP fluorescence did not significantly overlap. The absence of co-localization of Kv3.3 in GFP +ve neurones is in contrast to the reported co-expression of these two subunits and the potential for heteromultimeric *Shaw*-related potassium channel formation in rat spinal cord (Brooke et al., 2006). However, both subunits present unique patterns of expression in central neurones and the presence in thoracic (Brooke et al., 2006) or lumbar (this study) spinal cord of single-labelled Kv3.1b or Kv3.3 infers the putative existence of homomeric Kv3 channels. An association between Kv3.1b and GAD67-GFP expressing neurones has been demonstrated for both the medial septum diagonal band (Henderson et al., 2010) and cerebellar nuclei (Alonso-Espinaco et al., 2008) but to our knowledge this has not previously been reported for GAD65 GABAergic neurones or in spinal cord dorsal horn. However, given the limitations of immunofluorescence, electron microscopy studies will be required to fully determine the extent to which Kv3 subunits and GAD65 or GAD67 synthesizing enzymes co-exist in dorsal horn neurones.

In terms of physiology, by enhancing membrane repolarization and limiting after-hyperpolarization duration, Kv3 subunits confer on neurones a fast-spiking phenotype such that firing frequencies of 200–400 Hz are achieved and maintained for several seconds (Erisir et al., 1999). However, there are certainly examples of Kv3.1b expressing cells where a fast-spiking phenotype with such high frequencies are less evident (Dallas et al., 2005; Deuchars et al., 2001; Shevchenko et al., 2004). Electrophysiological analyses of GAD67-GFP interneurones in mouse lamina II (Heinke et al., 2004) revealed a degree of heterogeneity in recorded firing patterns although the most dominant was described as ‘initial bursting’ in which a short volley of action potentials occurred at the onset of depolarization. However, other studies have reported that a tonic firing pattern, where action potentials fire throughout the duration of depolarization, is more common in interneurones of superficial laminae expressing GAD67 (Daniele and MacDermott, 2009) or GAD65-GFP (Labrakakis et al., 2009). The physiological importance of specific neuronal firing patterns is unknown but neurones that are able to follow high frequencies may operate as ‘coincidence detectors’ or ‘high-pass filters’ whereas other neurone types act as ‘low-pass-filters’ or ‘integrators’ (Schneider, 2003; Prescott and DeKoninck, 2002). Speculatively, these biophysical properties allow for the control of transmission of high- and low-threshold primary afferent inputs by DH neurones in ways that are, as yet, poorly understood. In forebrain, many so-called fast-spiking interneurones that express Kv3 subunits have a parvalbumin phenotype (PV) (Chow et al., 1999). PV-containing cells, a proportion of which are GABAergic, are found in laminae II–III of the rat DH (Laing et al., 1994). An association between PV and GABA has been reported for GAD67-GFP transgenic mice (Dougherty et al., 2009) but the population as a whole was phenotypically quite diverse. These findings taken together with the fact that not all GAD65- or GAD67-GFP +ve interneurones examined here expressed either Kv3.1b or Kv3.3 suggest that these GABAergic cells present as a heterogeneous population. This is borne out by the mixed neurochemical phenotype and morphological diversity of GAD65 or GAD67 expressing DH interneurones seen in other transgenic mouse strains (Heinke et al., 2004; Labrakakis et al., 2009).

The extent to which these populations of GABAergic interneurones may be recruited after noxious peripheral stimuli was evaluated for both GAD65- and GAD67-GFP (Δneo) mouse strains. Expression of c-*fos* or Fos protein has been used extensively as an indicator of neuronal activation and the patterns of expression in response to a variety of noxious stimuli have been described (Williams et al., 1990; Bullitt, 1990). Capsaicin-induced noxious chemical stimulation of the hindpaw elevated the expression of c-*fos* in the DH of −/− littermates, GAD65- and GAD67-GFP mice with similar distribution patterns across DH laminae. In accord with previous studies of rat spinal cord DH, the highest increases in c-*fos* expression were associated with the ipsilateral superficial DH laminae although some augmentation was observed in the contralateral DH (Hunt et al., 1987; Jinks et al., 2002). Co-localization of GFP and c-*fos* expression was evident in the GAD65-GFP strain, albeit at a modest level of ∼20–30%. Significantly lower levels of co-localization were calculated for all laminae in GAD67-GFP (Δneo) mice. These data suggest differential recruitment of GAD65- or GAD67-expressing populations in response to peripheral short-lasting chemical nociception. Further studies will be required to determine relative levels of recruitment of GAD65- or GAD67-expressing populations in response to acute mechanical or thermal nociceptive stimuli not tested here. With respect to models of tonic pain, induction of Fos in GABAergic interneurones of rat DH in response to formalin has been profiled (Todd et al., 1994) and revealed that of the total population expressing Fos ∼20% of neurones were GABA-immunoreactive, some of these were also glycine-immunoreactive. Comparative analyses of c-*fos* in glycine and/or GABAergic containing neurones in acute and chronic pain models indicated significant activation of inhibitory interneurones though for the latter, c-*fos* expression was more abundant within laminae III–VI (Hossaini et al., 2010). These data taken together with the current finding of c-*fos* expression in mainly GAD65-GFP expressing interneurones indicates clearly that at least some inhibitory interneurones are recruited by noxious peripheral stimuli although the vast majority are not GABAergic. The receptive field properties of inhibitory neurones in DH are unknown but mono- and polysynaptic high threshold Aδ or C fibre drive onto inhibitory neurones within the DH has been described (Lu and Perl, 2003; Heinke et al., 2004; Hantman et al., 2004). A proportion of GABAergic interneurones, including the GAD67 +ve population, may also receive convergent inputs from low threshold Aβ afferents (Daniele and MacDermott, 2009). Spinal disinhibition is proposed to pathologically enhance pain sensitivity thereby contributing to hyperalgesia or allodynia (Sivilotti and Woolf, 1994; Zeilhofer, 2008). It is interesting that after neuropathic injury which elicits a reduction of GABAergic inhibition in neurones of LII there is a concomitant fall in ipsilateral levels of GAD65 but not GAD67 (Moore et al., 2002). Taken together these data suggest as yet unidentified and possibly distinctive roles for GAD65- and GAD67-expressing inhibitory neurones in the processing of afferent inputs and the response to peripheral nociception. Further physiological studies of these two populations are clearly required to clarify this more fully.
